# Enhancement of adoptive T cell transfer with single low dose pretreatment of doxorubicin or paclitaxel in mice

**DOI:** 10.18632/oncotarget.6628

**Published:** 2015-12-16

**Authors:** Fei-Ting Hsu, Tzu-Chun Chen, Hui-Yen Chuang, Ya-Fang Chang, Jeng-Jong Hwang

**Affiliations:** ^1^ Department of Biomedical Imaging and Radiological Sciences, National Yang-Ming University, Taipei, Taiwan; ^2^ Department of Medical Imaging, Taipei Medical University Hospital, Taipei, Taiwan; ^3^ Translational Imaging Research Center, College of Medicine, Taipei Medical University, Taipei, Taiwan; ^4^ Department of Radiation Oncology, Chang Gung Memorial Hospital, Linkou, Taiwan; ^5^ Biophotonics and Molecular Imaging Research Center (BMIRC), National Yang-Ming University, Taipei, Taiwan

**Keywords:** adoptive T cell transfer, bioluminescent imaging, doxorubicin, immunomodulation, NF-κB, paclitaxel, Immunology and Microbiology Section, Immune response, Immunity

## Abstract

*Ex vivo* expansion of CD8^+^ T-cells has been a hindrance for the success of adoptive T cell transfer in clinic. Currently, preconditioning with chemotherapy is used to modulate the patient immunity before ACT, however, the tumor microenvironment beneficial for transferring T cells may also be damaged. Here preconditioning with single low dose of doxorubicin or paclitaxel combined with fewer CD8^+^ T-cells was investigated to verify whether the same therapeutic efficacy of ACT could be achieved. An E.G7/OT1 animal model that involved adoptive transfer of OVA-specific CD8^+^ T-cells transduced with a granzyme B promoter-driven firefly luciferase and tomato fluorescent fusion reporter gene was used to evaluate this strategy. The result showed that CD8^+^ T-cells were activated and sustained longer in mice pretreated with one low-dose Dox or Tax. Enhanced therapeutic efficacy was found in Dox or Tax combined with 2×10^6^ CD8^+^ T-cells and achieved the same level of tumor growth inhibition as that of 5×10^6^ CD8^+^ T-cells group. Notably, reduced numbers of Tregs and myeloid derived suppressor cells were shown in combination groups. By contrast, the number of tumor-infiltrating cytotoxic T lymphocytes and IL-12 were increased. The NF-κB activity and immunosuppressive factors such as TGF-β, IDO, CCL2, VEGF, CCL22, COX-2 and IL-10 were suppressed. This study demonstrates that preconditioning with single low dose Dox or Tax and combined with two fifth of the original CD8^+^ T-cells could improve the tumor microenvironment *via* suppression of NF-κB and its related immunosuppressors, and activate more CD8^+^ T-cells which also stay longer.

## INTRODUCTION

Adoptive T cell transfer (ACT) using tumor-specific CD8+ T-cells are selected with specific tumor-associated antigens, expanded and activated with cytokines including interleukin (IL)-2, -7 and -15, then transfused back to the patient [1, 2]. However, ACT is currently not applied widely in clinic since the *ex vivo* large-scale expansion of T cells is inefficient and costly [3, 4]. Moreover, cancer cells may evolve and exert capabilities against attacks from transferred CD8+ T-cells, i.e. cytotoxic T lymphocytes, CTLs [5]. Hence, it is imperative to find a strategy to enhance the functions while reduce the required numbers of transferred CD8+ T-cells for ACT.

Cancer cells keep evolving during progression and could escape from immune surveillance. Immunosuppressive cytokines such as transforming growth factor-β (TGF-β) could inhibit the activation of CD8+ T-cells, which play the key role in hindrance of cancer elimination by ACT [6]. TGF-β also decreases expressions of anti-tumor cytokines including interferon gamma (IFN-γ) and interleukin-12 (IL-12), and further restrains proliferation and differentiation of T cells [7, 8]. The secretions of IFN-γ and IL-12 are also inhibited by IL-10 to impair the function of CD8+ T-cells [9]. Moreover, secretion of TGF-β, IL-10, chemokine (CC motif) ligand 22 (CCL22), cyclooxygenase-2 (COX-2), vascular endothelial growth factor (VEGF) and chemokine (C-C motif) ligand 2 (CCL2) will recruit immune regulatory cells such as regulatory T cells (Tregs) and myeloid derived suppressor cells (MDSCs) into tumor lesions [10-12]. Accumulation of these cells will suppress the functions and proliferation of CD8+ T-cells [13]. Furthermore, IDO expressed by cancer cells converts tryptophan into kynurenine which inhibits the proliferation of T cells and hinder the conversion of Tregs into T_H_17 [14, 15]. Expressions of CCL2, COX-2 and VEGF are also related to invasion, metastasis and angiogenesis [16-18].

Nuclear factor kappa-light-chain-enhancer of activated B cells (NF-κB), a transcription factor, has been shown to play a key hub for immune regulations [19-21]. NF-κB may promote the tumor formation and progression through up-regulation of its downstream effectors including TGF-β, IL-10, CCL2, COX-2, VEGF and CCL22 [22, 23]. Cancer cells could escape from the immune surveillance with expressions of these proteins. Thus targeting NF-κB may be an achievable strategy to modify the immunosuppression of tumor microenvironment [24]. Some chemotherapeutic or targeted drugs have been proposed to trigger antitumor immunity other than eliminate cancer cells directly [25]. Our recent findings suggest that serial low doses of curcumin or sorafenib combined with ACT exhibit better tumor inhibition [26, 27]. However, it has been reported that Dox and Tax may induce the activation of NF-κB under the administration of clinical dosage [28-30]. Thus, preconditioning using optimal dosage of Dox or Tax to avoid activating NF-κB and its downstream effectors is critical for the success of ACT. Here we aimed to investigate whether single low dose of Dox or Tax prior to ACT could augment the treatment outcome and the related underlying mechanisms. The *pGBeLT* reporter system established by Patel et al. to monitor the activation of transferred CD8+ T-cells for ACT in E.G7/OT-1 mouse model was used [31]. Immunosuppressive cytokines and other immune cells such as Tregs and MDSCs were also determined.

## RESULTS

### Immunosuppressive factors are suppressed by one low-dose Dox or Tax through reducing NF-κB activity in E.G7 cells

**Figure 1:**
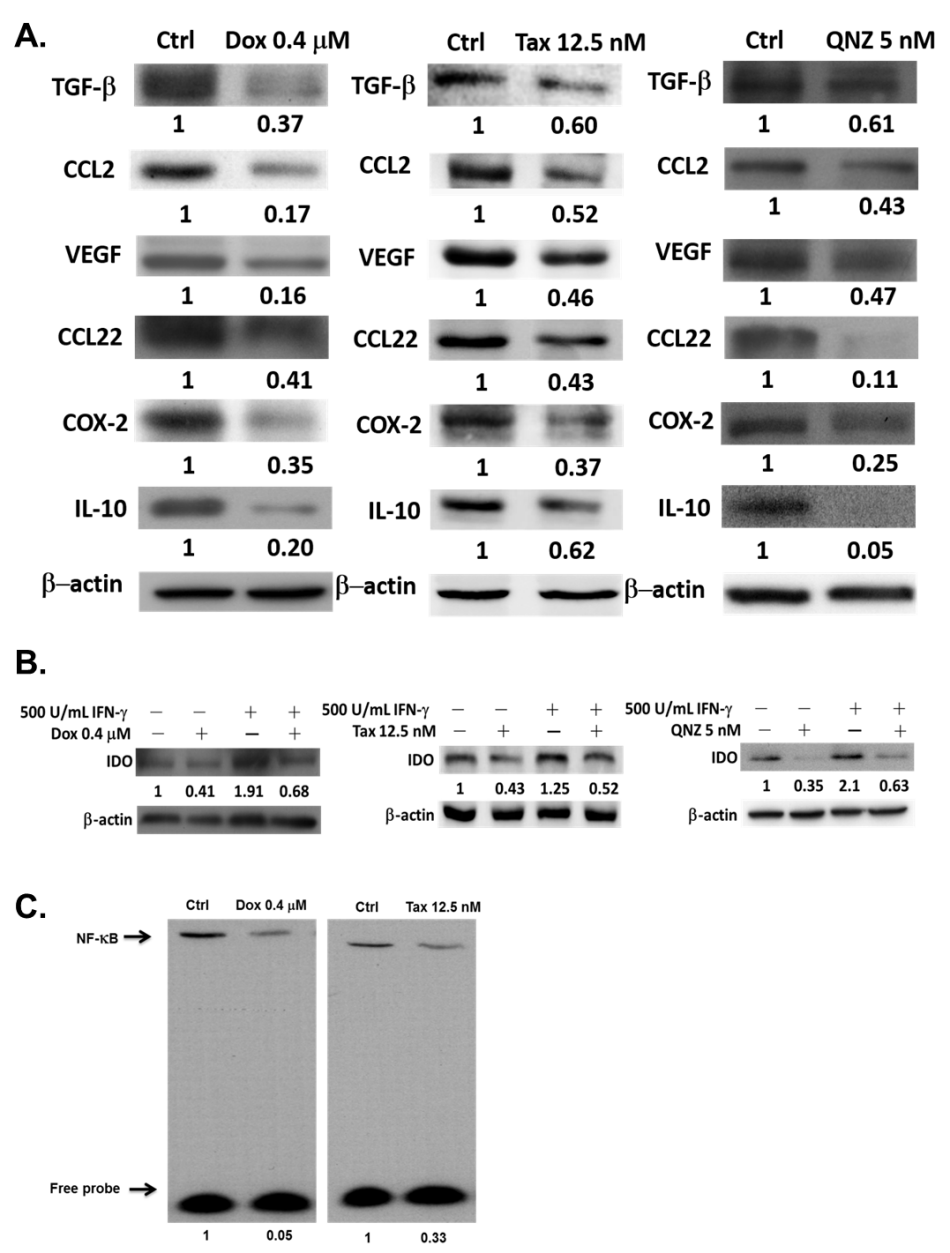
Expressions of immunosuppressive factors are decreased by one low-dose Dox or Tax *via* inhibition of NF-κB pathway **A.** Proteins were extracted from E.G7 cells treated with 0.4 μM Dox, 12.5 nM Tax and 5 nM QNZ (NF-κB inhibitor, QNZ), respectively, for 24 hours, and assayed by Western blot. Expressions of immunosuppressive factors were decreased in a dose-dependent manner. **B.** Both 0.4 μM Dox and 12.5 nM Tax could inhibit the expression of exogenous IDO stimulated by IFN-γ. 5 nM QNZ was the positive control. **C.** NF-κB activities in E.G7 cells treated with 0.4 μM Dox and 12.5 nM Tax, respectively, for 24 hours were significantly inhibited according to EMSA assay.

The survivals of E.G7 mouse lymphoma cells treated with different concentrations of Dox, Tax and QNZ, a NF-κB inhibitor, respectively, were shown in Supplementary Figure 1A-1C. The expressions of TGF-β, CCL2, VEGF, CCL22, COX-2 and IL-10 in E.G7 cells were significantly suppressed by 0.4 μM Dox, 12.5 nM Tax and 5 nM QNZ (Figure 1A). To mimic the tumor microenvironment, 500 U/mL IFN-γ was added into the cultured medium to stimulate the expression of IDO, an enzyme often overexpressed in the tumor microenvironment. IDO was highly expressed after IFN-γ stimulation, but was suppressed by Dox (0.4 μM), Tax (12.5 nM) and QNZ (5 nM), respectively (Figure 1B). Notably, significant inhibitions of NF-κB/DNA binding activity by Dox and Tax were also found (Figure 1C).

### Enhancement of CD8+ T-cells activation and migration by one low-dose Dox or Tax is through downregulation of NF-κB activation in E.G7 cells

**Figure 2:**
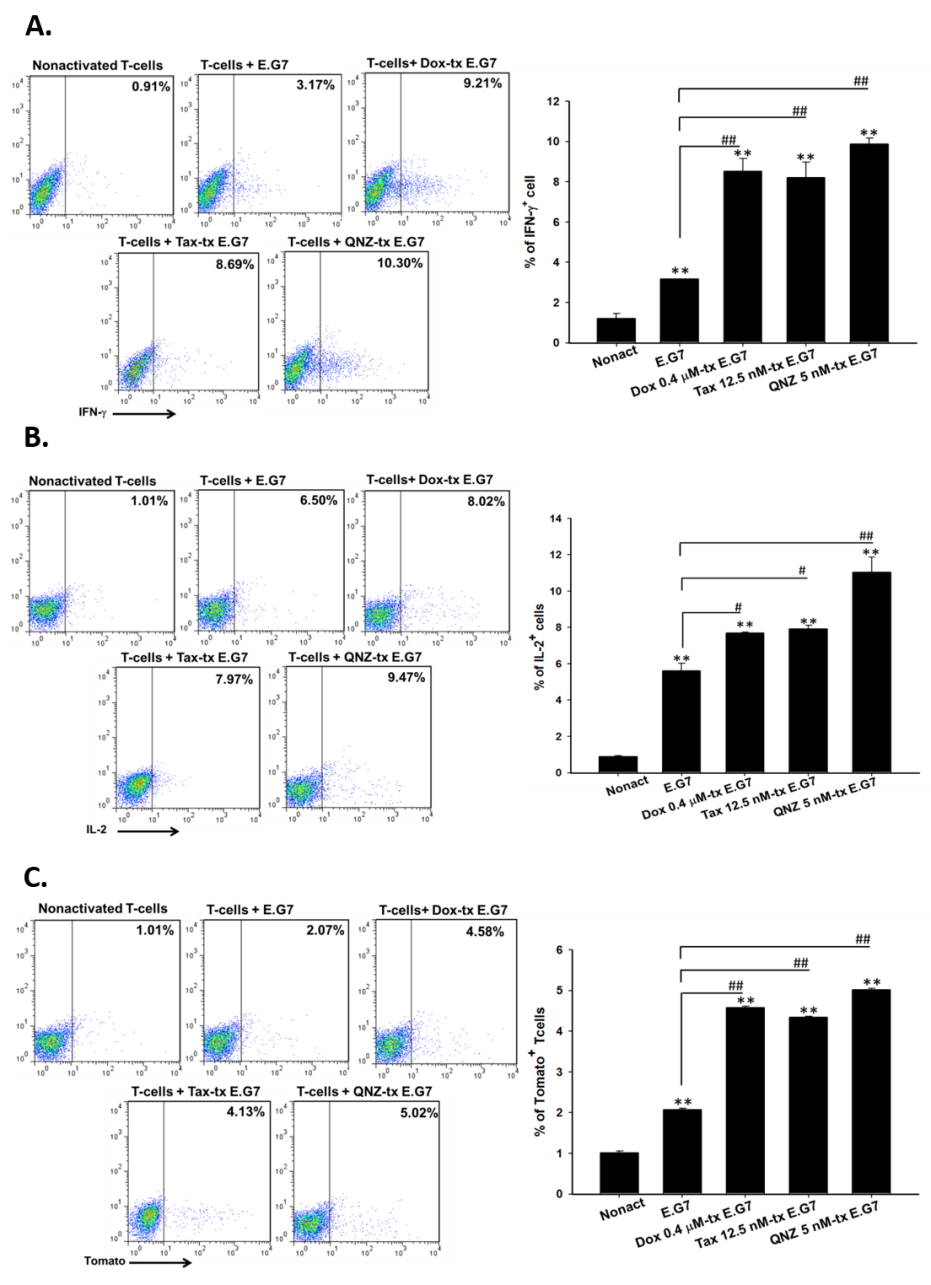

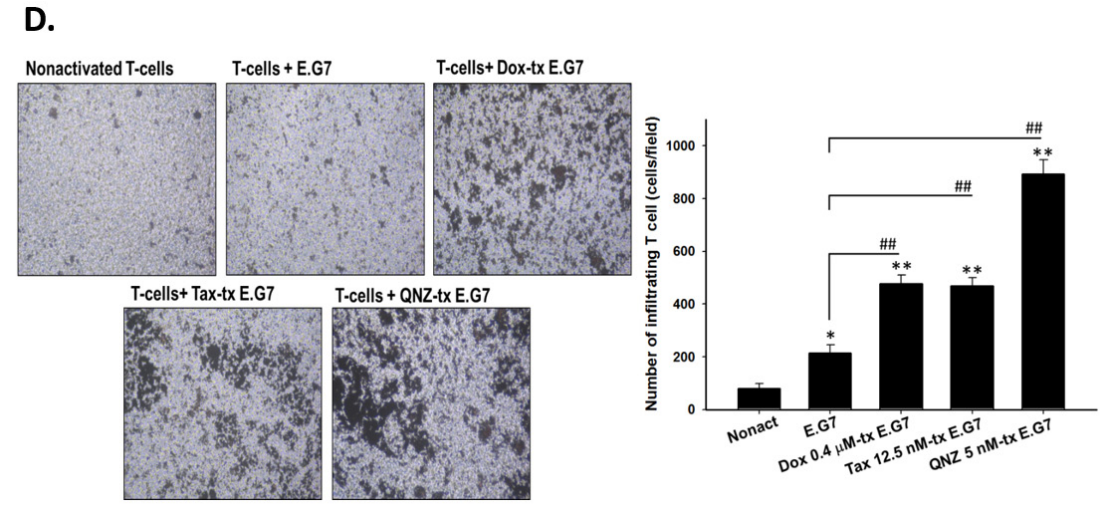
Low-dose Dox or Tax enhanced activation and migration of CD8+ T-cells through inhibition of NF-κB **A.**-**B.** Isolated CD8+ T-cells were co-cultured with E.G7 cells pretreated with 0.4 μM Dox, 12.5 nM Tax and 5 nM QNZ, respectively, at the effector-to-target ratio 25. Intracellular IFN-γ and IL-2 were significantly increased in the Dox, Tax and QNZ-pretreated groups compared with the untreated group. **C.** Expression of tomato fluorescent protein in *pGBeLT*-transduced CD8+ T-cells was evaluated, and was found significantly increased in all pretreated groups. **D.** Migratory CD8+ T-cells were remarkably increased in the groups pretreated with low-dose Dox, Tax or QNZ. (* *p* < 0.05, ** *p* < 0.01, * compared with that of non-activated group; ^#^ compared with that of untreated E.G7 group)

The percentages of intracellular IFN-γ-, IL-2- and tomato fluorescence-positive CD8+ T-cells were increased significantly in low-dose Dox, Tax and QNZ-pretreated groups compared with that of vehicle (Figure 2A-2C), suggested that low-dose Dox, Tax and QNZ could enhance the activation of CD8+ T-cells. The transwell assay also showed that the numbers of migrated T cells were increased by the treatments of low-dose Dox, Tax and QNZ (Figure 2D).

### Single low dose Dox or Tax suppresses expressions of immunosuppressive factors through inhibition of NF-κB activity

**Figure 3:**
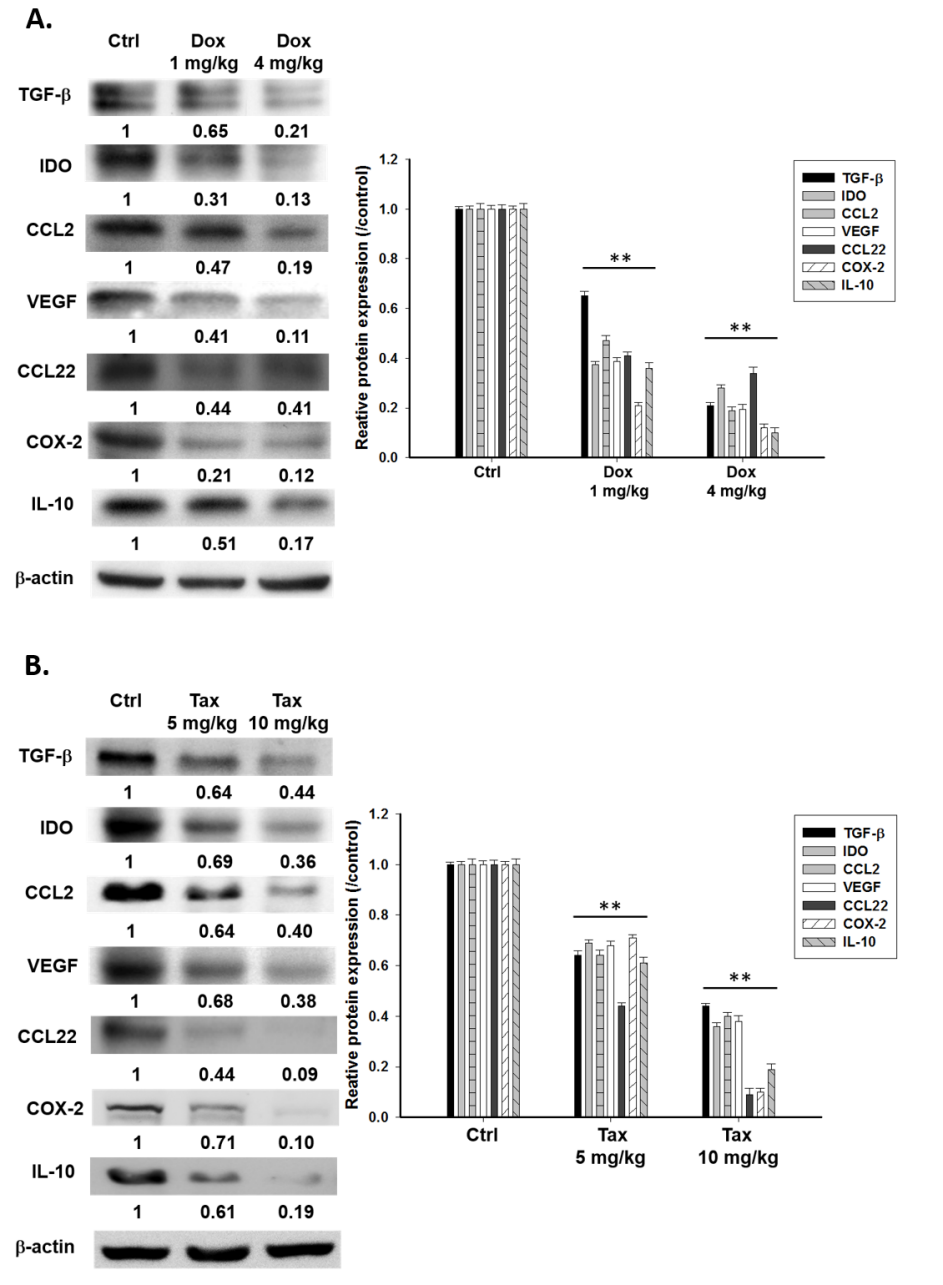

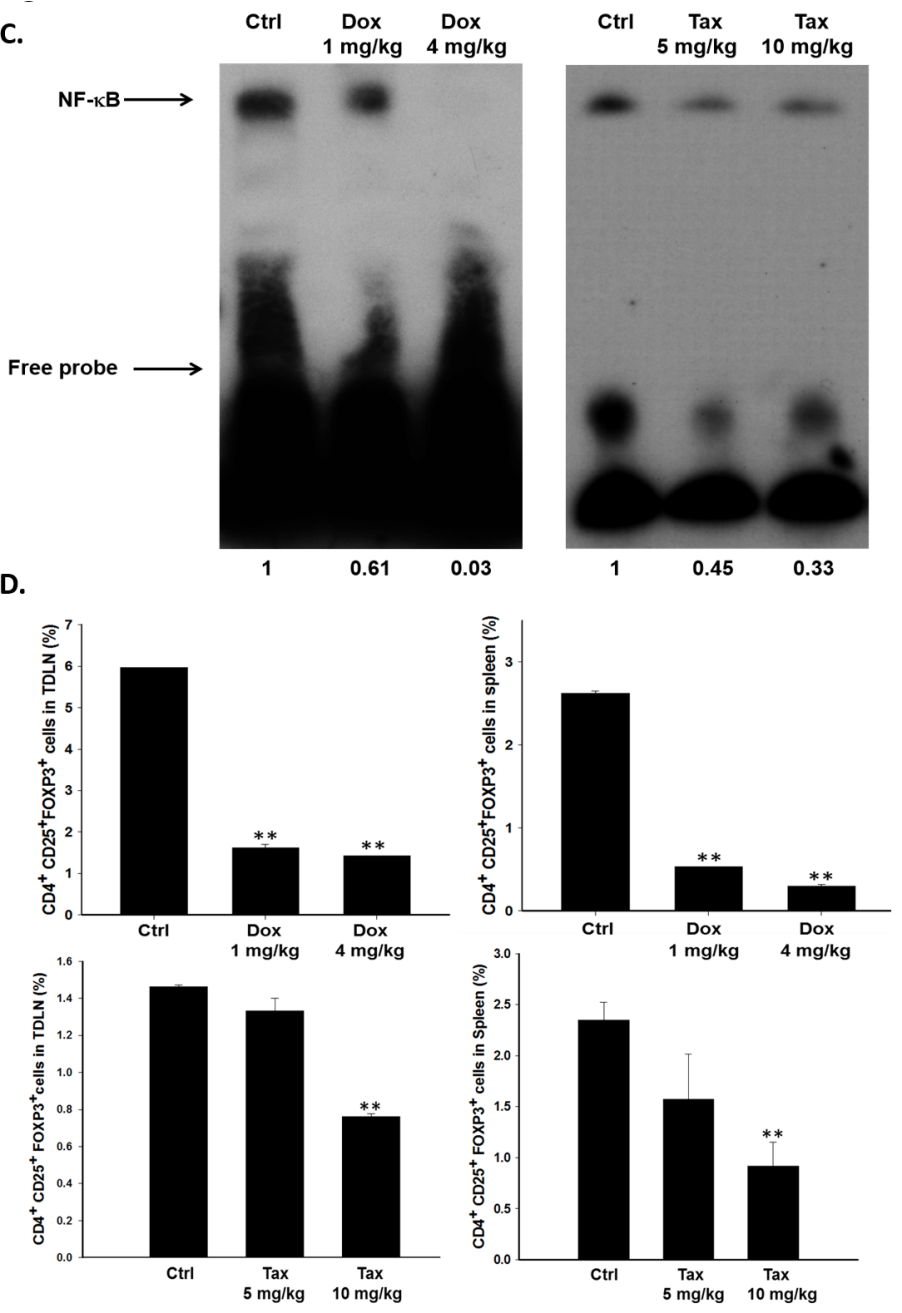

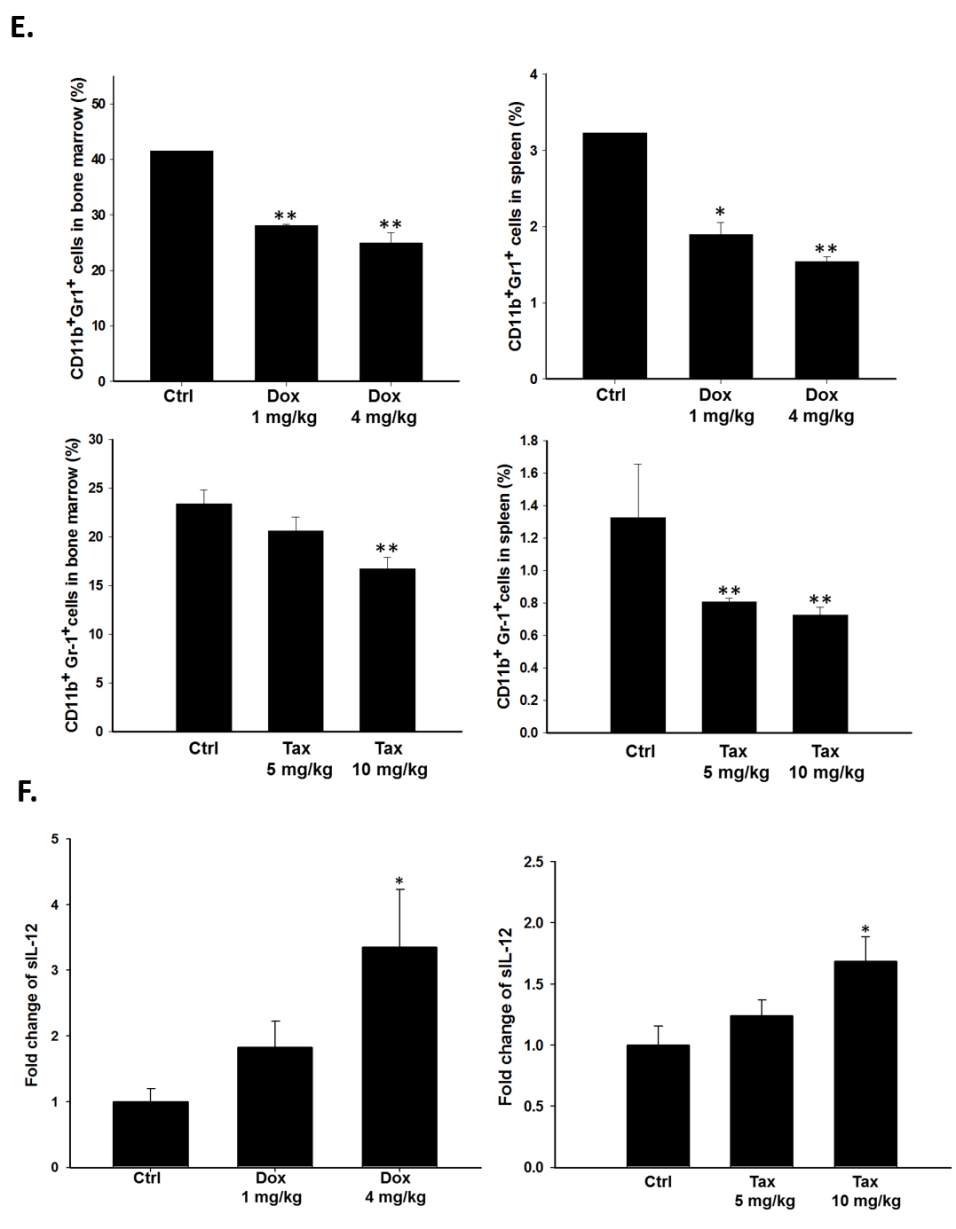
Immunosuppressive factors and cells were suppressed *via* inhibition of NF-κB three days post single low dose treatment of Dox (1 and 4 mg/kg) and Tax (5 and 10 mg/kg), respectively The E.G7 tumor-bearing mice were established on day -7 and treated with or without single low dose of Dox or Tax on day -1 (tumor volume about 100 mm^3^) followed by ACT on day 0. All mice were sacrificed on day 3. **A.**-**B.** Proteins extracted from tumors of Dox- or Tax-treated mice were analyzed with Western blotting. TGF-β, IDO, CCL2, VEGF, CCL22, COX-2 and IL-10 were significantly decreased in a dose-dependent manner. **C.** Nuclear proteins extracted from tumors of mice of each group were analyzed with EMSA. NF-κB/DNA binding activity was decreased by single low dose treatment of Dox and Tax, respectively, in a dose-dependent manner. **D.** Percentages of Tregs in TDLN and spleen were significantly decreased in mice treated with single low dose of Dox or Tax. **E.** Percentages of MDSCs in bone marrow and spleen were significantly decreased in mice treated with single low dose of Dox or Tax. **F.** IL-12 levels in the serum assayed by ELISA were increased in a dose-dependent manner in mice treated with single low dose of Dox or Tax. (* *p* < 0.05, ** *p* < 0.01 compared with that of the control).

E.G7 tumor-bearing mice were established on day -7 and treated with or without single low doses of Dox (1 and 4 mg/kg) or Tax (5 and 10 mg/kg) on day -1, followed by ACT on day 0, and mice were sacrificed on day3. The expressions of TGF-β, IDO, CCL2, VEGF, CCL22, COX-2 and IL-10 in tumors were significantly reduced in Dox- and Tax-treated groups (Figures 3A and 3B). The results were similar with those found *in vitro*. Notably, these immunosuppressive factors were decreased starting from day 1 post single low dose Dox and Tax treatment, respectively, as shown in Supplementary Figure 2A and 2B. The NF-κB/DNA binding activity in tumors was suppressed in both Dox- and Tax-treated groups as demonstrated with EMSA (Figure 3C).

### Single low-dose Dox or Tax reduces the percentages of regulatory T cells and myeloid derived suppressor cells in mice

The percentages of Tregs (CD4^+^CD25^+^FoxP3^+^) and MDSCs (CD11b^+^Gr1^+^) in TDLN/spleen and bone marrow/spleen, respectively, were significantly decreased after single low-dose Dox or Tax treatment (Figures 3D and 3E). Significant decreases of these immunosuppressors were also found on day 1 post one low-dose Dox or Tax treatment as shown in Supplementary Figure 2C and 2D. On the contrary, the levels of serum IL-12 were increased on day 3 post one low-dose Dox or Tax treatment determined with ELISA (Figure 3F).

### Therapeutic efficacy of ACT is improved by pretreatment with single low dose Dox or Tax

**Figure 4:**
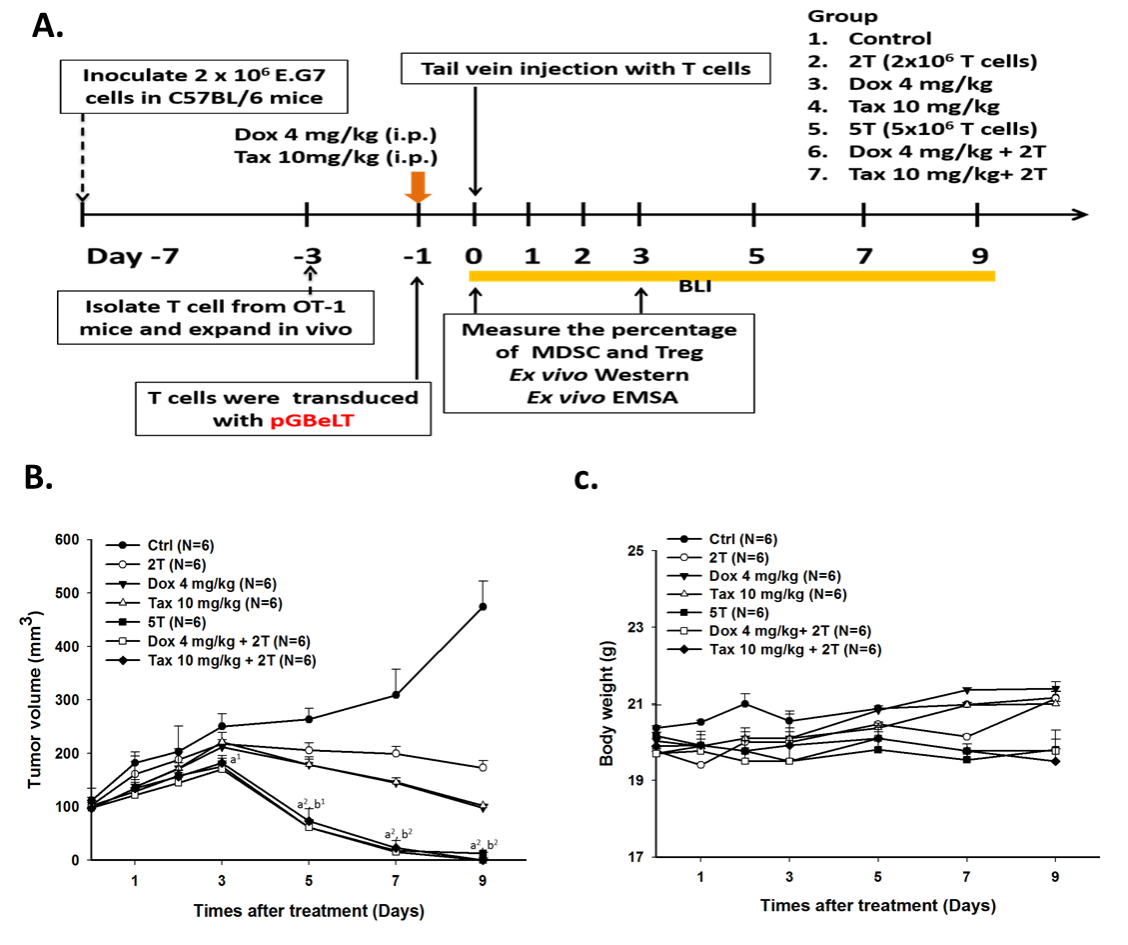

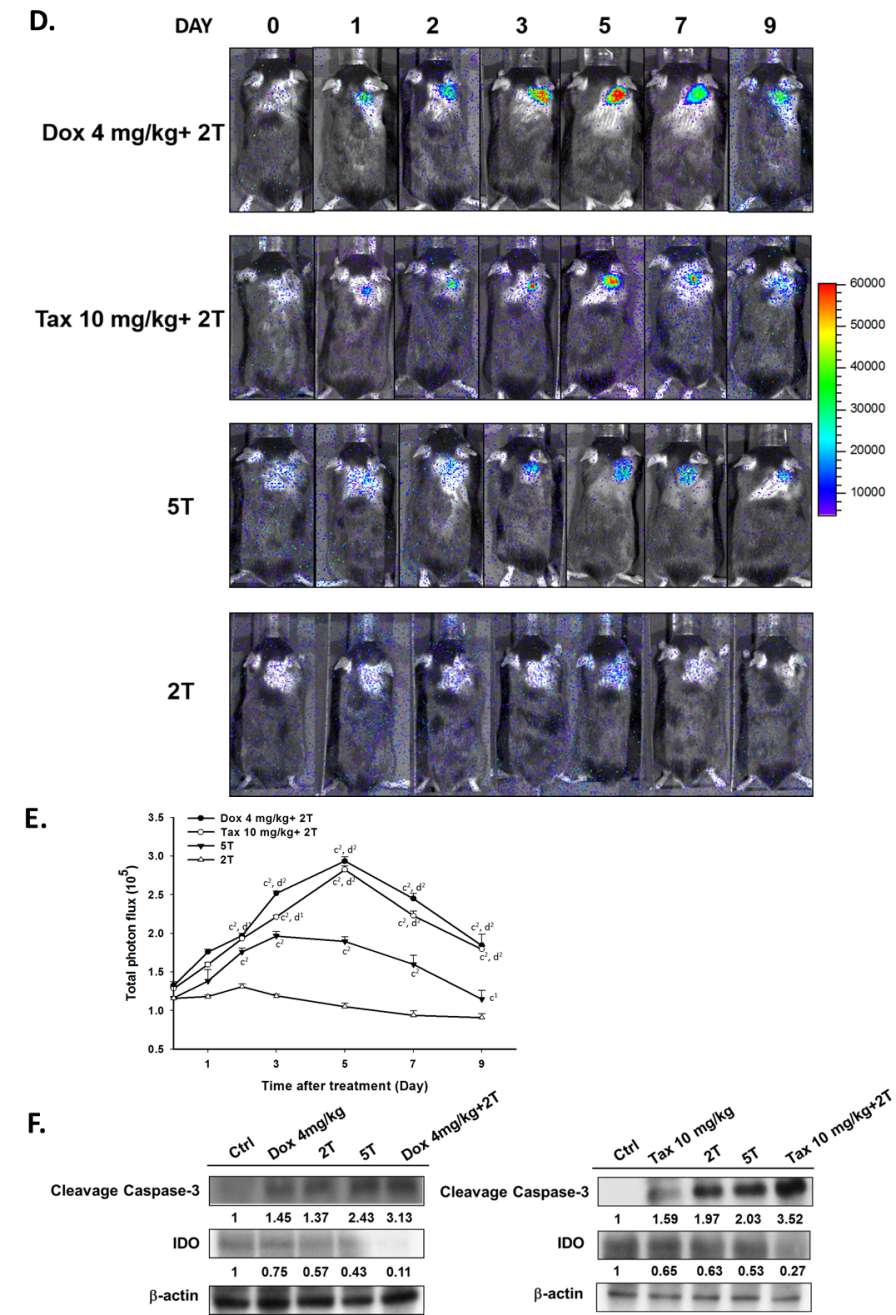
Therapeutic efficacy was enhanced by single low-dose Dox or Tax combined with ACT **A.** the experimental design *in vivo*. Mice were randomly separated into seven groups as shown in figure. The ACT was performed on day 0, and single low-dose Dox or Tax was given one day before ACT (i.e. D-1). CD8+ T-cells activations were monitored by BLI; furthermore, immunosuppressive cells were also analyzed by flow cytometry. **B.** tumor volumes were measured by digital caliper daily. Tumors in the two combinational groups and 5T group started to shrink markedly from day 3 post ACT. **C.** body weight was tracked to assess the general toxicities of treatments. (a *vs*. Ctrl, b vs. 2T, ^1^*p* < 0.05 and ^2^*p* < 0.01) **D.** *pGBeLT*-transduced CD8+ T-cells receiving mice were imaged with IVIS50 imaging system to monitor the activations of transferred CD8+ T-cells. **E.** the quantification of bioluminescent signals emitted from transferred CD8+ T-cells. (c vs. 2T, d vs. 5T, ^1^*p* < 0.05 and ^2^*p* < 0.01) **F.** the expressions of cleavage caspase-3 were increased in all treated groups, and the highest expressions were found in the two combinational groups. On the contrary, the IDO expression was decreased after treatments. *n* = 6 per group.

Since we have found that 70 mg/kg/d curcumin combined with 5×10^6^ OT-1 CD8+ T-cells could completely inhibit the growth of E.G7 tumor, 5×10^6^ OT-1 CD8+ T-cells were also used here [27]. E.G7 tumor-bearing mice were randomly separated into seven groups (*n* = 6 per group) as described in the method when the average tumor size reached about 100 mm^3^. Single low dose Dox (4 mg/kg) or Tax (10 mg/kg) was injected intraperitoneally prior to ACT, and was designated as day -1. The entire experimental protocol was shown in Figure 4A. Significant tumor shrinkage was found in two combination groups (i.e. Dox+2T and Tax+2T) on day 3 post ACT compared with that of the control group (Figure 4B). Notably, the tumor growth inhibition of combination groups were the same compared with that of 5T group (T stands for 1 × 10^6^ OT-1 CD8+ T-cells). No significant body weight change was found in all groups throughout the experiment (Figure 4C).

A granzyme B promoter-driven firefly luciferase and tomato fluorescent fusion reporter gene (*pGBeLT*) system was used to monitor the activation and distribution of transferred OT-1 CD8+ T-cells. The baseline images were acquired at 4 hours after ACT, and the activation of OT-1 CD8+ T-cells were monitored by BLI at designated time points as shown in Figure 4D. The signals emitted from tumors were increased and peaked on day 5 post ACT for both combination groups, and were significantly higher than those of 5T and 2T groups, respectively (Figure 4E). The levels of cleaved caspase-3 in tumors were most increased, by contrast, IDOs were severely decreased on day 3 post ACT in two combination groups assayed with Western blot (Figure 4F).

### Ratios of tumor-infiltrating CD4^+^FoxP3^+^ Tregs/CD8^+^ CTLs are decreased in experimental groups

**Figure 5:**
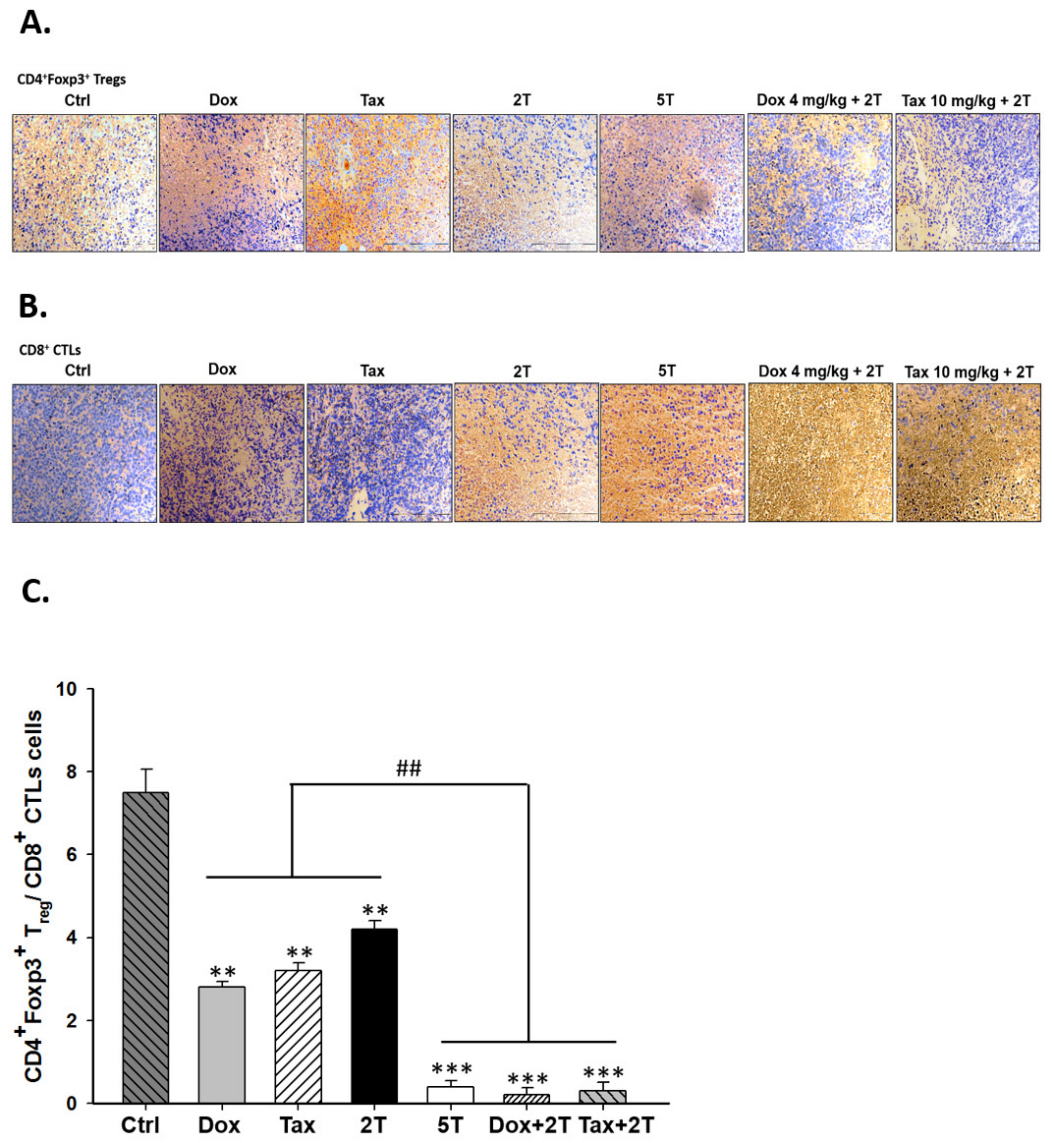
Single low-dose Dox or Tax increased the numbers of tumor-infiltrating CD8+ T-cells but not CD4+CD25+FOXP3+ Tregs in the combinational groups **A.** tumor-infiltrating FOXP3+ cells were significantly lower in all treatment groups than that of the control group. **B.** CD8+ T-cells were found increased in the two combinational groups compared with the single treatment counterparts. **C.** ratios of FOXP3+ Tregs/CD8+ CTLs were calculated, the ratios were strikingly decreased in the combinational groups. (** *p* < 0.01, ** *p* < 0.005, * compared with that of control group; ^##^ *p* < 0.01, ^#^ compared with that of single treatment group).

From immunohistochemical analysis, tumor-infiltrating CD4^+^FoxP3^+^ Tregs were decreased while tumor-infiltrating CTLs were increased in all experimental groups especially in combination groups compared with those of the control group (Figures 5A and 5B). Accordingly, ratios of CD4^+^FoxP3^+^ Tregs/CD8^+^ CTLs were significantly decreased in two combination groups compared with that of the control or drug alone (Figure 5C).

## DISCUSSION

Large scale *ex vivo* expansion of tumor-specific T cells and the immunosuppressive tumor microenvironment have been the hindrance for clinical application of ACT [3, 4]. The numbers, survival and activities of transferred CD8+ T-cells are factors affecting the tumor cell killing *in situ* during ACT [32]. To reverse the immunosuppressive tumor microenvironment may be one of the most challenging issues in cancer immunotherapy [33]. Some “off-target” effects of chemotherapeutic drugs on innate and adaptive immunity rather than cytotoxicity have been found [25, 34]. Better tumor responses were also found in the groups received both chemotherapy and ACT [27, 35, 36]. However, myelosuppression induced by chemotherapy limits the clinical use of both Dox and Tax [37]. Here we found that single low dose pretreatment of Dox or Tax could improve the immunosuppressive tumor microenvironment through increasing the number, activity and functions of tumor-infiltrating transferred CD8+ T-cells in an E.G7/OT-1 mouse model. Hence, this strategy might be a feasible approach to improve the therapeutic efficacy of ACT. The doses of Dox and Tax used in this study are defined as “low-dose” compared with the clinical dosages based on the formula proposed by Reagan-Shaw *et al.* [38]. According to the formula, 1-4 mg/kg Dox and 5-10 mg/kg Tax in this study were equivalent to 3-12 mg/m^2^ Dox and 15-30 mg/m^2^ Tax in clinic.

The expressions of immunosuppressive factors in E.G7 cells including TGF-β, CCL2, VEGF, CCL22, COX-2 and IL-10 were decreased after treatment with low-dose Dox (0.4 μM) or Tax (12.5 nM) as shown in Figure 1A. CCL2, CCL22, TGF-β and IL-10 augment the differentiation of M2-like tumor-associated macrophages (TAMs) and recruitment of immunosuppressors including TAMs, MDSCs and Tregs [39]. These immunosuppressors secret TGF-β, IL-10 and other chemokines, result in more immunosuppressive tumor microenvironment. CCL2 has been shown to be pivotal for immunosuppression, and the CCL2/CCR2 pathway could be a potential target for cancer therapy [16, 17]. IDO which catalyzes tryptophan into kynurenine is overexpressed by cancer cells to function as an immunosuppressive factor. The expressions of both endogenous and exogenous IDOs were suppressed by low-dose Dox or Tax (Figure 1B). TGF-β also increases IDO expression through activating non-conical NF-κB pathway [40]. The NF-κB/DNA binding activity detected by EMSA assay was significantly inhibited by low-dose Dox and Tax (Figure 1C). According to these results support that low-dose Dox or Tax may function as a NF-κB inhibitor. NF-κB may function as a substantial regulator in tumor microenvironment. Activated NF-κB in tumor cells results in high expressions of immunosuppressive factors such as IL-6, IL-10, TGF-β and VEGF, and impedes the anti-tumor immunity [19, 41]. Since the functions and proliferation of transferred CD8+ T-cells could be impaired by immunosuppressive microenviroment [10], here co-culture of isolated CD8+ T-cells and QNZ (a NF-B inhibitor) pretreated E.G7 cells showed that the most activation (IFN-γ, IL-12, Tomato^+^ T cells) and migration (infiltrating T cells) of CD8+ T-cells were found (Figures 2A-2D). These results conclude that inhibition of NF-κB may enhance the activation and migration of transferred CD8+ T-cells. Our animal studies further confirm these findings. NF-κB/DNA binding activity and immunosuppressive factors were decreased in tumors of mice pretreated with single low-dose Dox or Tax (Figures 3A-3C). The numbers of Tregs and MDSCs in TDLNs, spleen and bone marrow were found less accumulated (Figures 3D and 3E). Recently, Loxoribin, a TLR7 agonist, has been shown to be able to shrink the tumor through augmenting the proliferation of CD4+ T cells and lowering the frequency of Tregs, which is mediated by elevated IL-6 level secreted by DCs. [42]. IL-12 has been shown with anti-tumor effect and could enhance expressions of other cytokines involving in both innate and adaptive immunity [43]. The serum levels of IL-12 in mice were increased in a dose-dependent manner after pretreatments of low-dose Dox or Tax suggesting that the function of CD8+ T-cells might be enhanced *via* IL-12 regulation (Figure 3F). Together, our *in vitro* and *in vivo* results suggest that the therapeutic efficacy of ACT could be improved when pretreated with single low dose of Dox or Tax.

Recently, the outcome of ACT in breast cancer has been shown to be enhanced by low-dose Dox (5 mg/kg *i.v.*) plus Th_1_ or Th_17_ cells (*i.v.* or intratumoral), which results in increased proliferations of CD8+ T-cells and NK cells through elimination of MDSCs [43]. Here we demonstrated that single low-dose Dox (4 mg/kg *i.p.*) or Tax (10 mg/kg *i.p.*) administered one day prior to ACT which used fewer numbers of CD8+ T-cells (i.e. Dox + 2 × 10^6^ T cells or Tax + 2 × 10^6^ T cells) could achieve the same tumor inhibition efficacy as that of 5 × 10^6^ T cells group. Since transferred CD8+ T-cells traffic indiscriminately and ubiquitously into various tissues/organs and may cause potential side effects [44], thus minor or neglected adverse effects related to transferred CD8+ T-cells could be avoided with this combinational strategy. The activation and survival of transferred CD8+ T-cells were improved in combinational groups assayed by *pGBeLT* system (Figures 4D and 4E). Moreover, the numbers of tumor-infiltrating CD8+ T-cells and Tregs have been reported to closely correlate with the prognosis and survival of patients [45, 46]. Alternatively, the expansion efficiency and the cell function also could be enhanced by the culturing strategy such as using the bone marrow multipotent mesenchymal stromal cells (BM-MMSC) [47]. Here we have shown that the activity and number of tumor-infiltrating CD8+ T-cells were strikingly increased while the number of tumor-infiltrating Tregs was diminished in combination groups (Figures 5A-5C). The expression of cleaved caspase-3 in tumors was also significantly increased in combination groups (Figure 5F), suggesting that tumor cell killing by CD8+ T-cells was through the apoptotic pathway.

**Figure 6:**
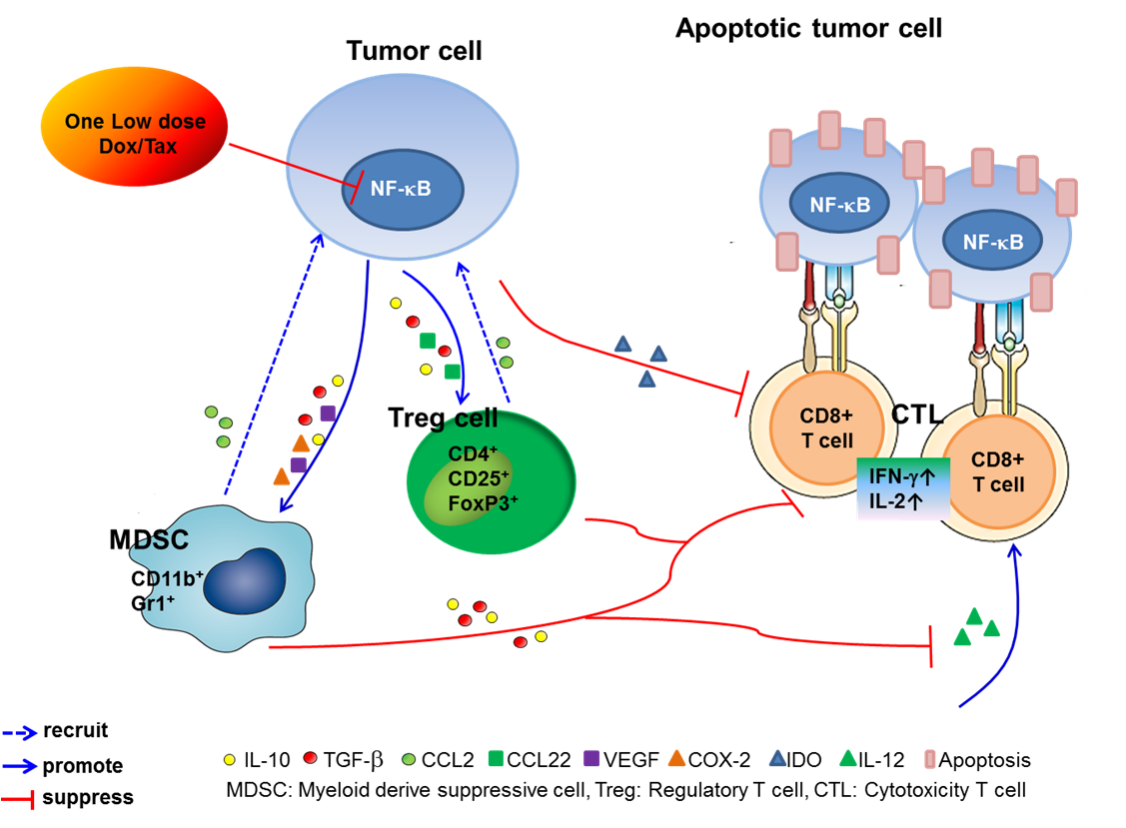
The proposed underlying mechanism for modulation of the immune tumor microenvironment and enhancement of the therapeutic efficacy of ACT in cancer pretreated with one low-dose Dox or Tax

In conclusion, our results suggest that tumor-bearing mice pretreated with single low-dose Dox or Tax may improve the therapeutic efficacy of ACT which using two millions, instead of five millions, of CD8+ T-cells for the treatment of cancer. The therapeutics of possible underlying mechanisms are the improvement of the tumor microenvironment through the suppression of NF-κB and subsequent cytokines such as TGF-β, IL-10, VEGF, COX-2, CCL2 and CCL22, and enhancement the function of transferred CD8+ T-cells (Figure 6). This combination strategy may be potential to overcome the current limitation of ACT in clinic.

## MATERIALS AND METHODS

### Cell culture and reagents

OVA-expressing E.G7 mouse lymphoma cell line and Jurkat human T-cell leukemia cell line purchased from American Type Culture Collection (ATCC; Jan 2010) were maintained in cRPMI-1640 (RPMI-1640, 10% FBS, 1% penicillin/streptomycin, and 25 mM HEPES) with 400 μg/mL G418. Lentiviral-producing HEK-293FT cell line from Invitrogen (Jan 2010) was maintained in cDMEM [Dulbecco's modified Eagle's medium (DMEM), 10% FBS, 1% penicillin/streptomycin, 2 mM L-glutamine, and 0.1 mM MEM nonessential amino acids] with 500 μg/ml G418. All cell lines were authenticated by short tandem repeat profiling prior to their usage and passed for less than 6 months in our experiments. Cell culture tested *Myacoplasm* free routinely with MycoAlert Mycoplasma Detection Assay (Lonza). Lentiviral production and titration assay were performed as previously described [26]. 6-Amino-4-(4-phenoxyphenylethylamino)quinazoline (QNZ, Millipore), a NF-κB inhibitor, was dissolved in 0.1% dimethyl sulfoxide (DMSO, Sigma) and prepared 1 mM as stock solution. Doxorubicin (Pfizer) was dissolved in 0.1% DMSO and prepared 1 mM as stock solution for *in vitro* studies. For *in vivo* studies, Dox was dissolved in 0.1% DMSO and prepared 10 mg/ml as stock solution. Paclitaxel (6 mg/ml/vial) was purchased from Yung Shin Pharm. Ind. Co., Ltd, Taiwan, and was prepared 10 μM as stock solution for *in vitro* study. All stock solutions were stored at −20°C. The doses used *in vivo* were 1 and 4 mg/kg for Dox and 5 and 10 mg/kg for Tax [37, 48], respectively.

### Animals

Eight-week-old male C57BL/6 mice purchased from the National Laboratory Animal Center, Taiwan and OT-1 transgenic mice obtained from the Jackson Laboratory were housed in the laboratory animal center of National Yang-Ming University, and maintained as previously described [27]. All animal protocols were approved by the Animal Care and Use Committee at National Yang-Ming University (permits number: 1000522). All animal experiment was followed by the UKCCCR guidelines for the welfare of animals in experimental neoplasia [49].

### Isolation and transduction of mouse CD8+ T-cells

Isolation and transduction were performed as previously described [26, 27]. In brief, CD8+ T-cells isolated from spleens of OT-1 mice were enriched by mouse T cell enrichment kit (Stemcell Technologies), then cultured in cRPMI-1640 medium containing 2.5 μg/ml Concanavalin A (Calbiochem), 50 μM β-mercaptoethanol (Bio-Rad), 10 ng/ml IL-7 (R&D Systems) for 36 hours. CD8+ T-cells were transduced with *pGBeLT* lentiviruses (MOI = 5) for 4 hours, and cultured in cRPMI-1640 medium containing 50 μM β-ME, 10 ng/mL IL-15 and 10 U/mL IL-2 (R&D Systems), and 50 mM α-methyl mannoside (Calbiochem) for another 24 hours.

### Cytotoxicity of Dox, Tax and NF-κB inhibitor on E.G7 cells

1×10^5^ E.G7 cells/well were cultured in 96-well plate and treated with various concentrations of Dox, Tax and QNZ, respectively, for 24 hours. Cytotoxicity was determined by AlamarBlue assay. Briefly, 10% AlamarBlue reagent containing cRPMI-1640 (product code: BUF012B, AbD Serotec) was added to cells and incubated at 37°C for 4 hours. The fluorescent signals were detected at 570 nm and 600 nm, respectively, using ELISA reader (TECAN Sunrise). Cell viability was calculated using the equation provided by the manufacture.

### Transwell assay

1×10^6^ E.G7 cells/well pretreated with or without 0.4 μM Dox, 12.5 nM Tax and 5 nM QNZ, respectively, were resuspended in 500 μl cRPMI-1640 and seeded into 24-well plates. CD8+ T-cells were resuspended in cRPMI-1640 at a density of 2×10^6^ cells/ml, and 100 μl of CD8+ T-cells suspension was added into the upper compartment of 5 μm transwell inserts (Corning Costar). After 4 hours incubation, the membranes were fixed, stained with hematoxylin and air-dried. The number of migratory CD8+T cells was photographed and counted under Leica DM IRB microscope (Deerfield) with 100x magnification, and five fields per slide were scored.

### Flow cytometric analysis

After 4 hours co-culture, the activations of CD8+ T-cells were determined by the expressions of tomato fluorescent protein, intracellular IFN-γ and IL-2 (Biolegend) using flow cytometry. For *in vivo* study, the tumor draining lymph nodes (TDLNs) and bone marrow from the vehicle, Dox- and Tax-treated mice were harvested and analyzed on day 3 post Dox and Tax administration to trace the percentages of Tregs and MDSCs (*n* = 6 per group). The single cell suspensions isolated from the spleen, TDLNs and bone marrow were stained with anti-FOXP3-Alexa Fluor 488/CD4-APC/CD25-PE antibodies using a Mouse Treg Flow Kit (Biolegend) according to manufacturer's protocol, and CD11b-FITC and Gr-1-PE antibodies (eBioscience) for detecting Tregs and MDSCs, respectively. The percentages of these cell types were acquired using FACSCalibur flow cytometry (BD Bioscience), and data were analyzed by FlowJo software (Tree Star).

### Western blotting

To assess the expressions of immunosuppressive factors, proteins were extracted from E.G7 cells or removed tumors using lysis buffer and T-PER kit (Thermo Scientific), respectively. For *in vitro* studies, 1×10^6^ E.G7 cells were seeded into each 10-cm diameter culture dish and treated with Dox, Tax and QNZ, respectively, for 24 hours prior to protein extraction. 500 U/ml IFN-γ (BD Biosciences) was added into the medium to stimulate E.G7 cells to evaluate whether Dox, Tax and QNZ could inhibit the expression of exogenous IDO. Protein lysates were separated by 10-15% SDS-PAGE, transferred to polyvinylidene difluoride membrane (Pall), blocked with 5% nonfat milk dissolved in TBST (Tris-buffered saline containing 0.1% Tween-20) at room temperature for 1 hour. The membranes were incubated with primary antibodies against anti-IDO, anti-COX-2 (Millipore), TGF-β, CCL2, (Cell Signaling), anti-MDC (CCL22), anti-VEGF, anti-IL-10 and β-actin (Abcam) at 4°C overnight. After washed with TBST, the membranes were incubated with horseradish peroxidase-conjugated secondary antibodies (Jackson ImmunoResearch Laboratories Inc.) for 1 hour at room temperature. Finally, the proteins of interest were visualized by the Image Quant LAS-4000 (Fujifilm) with the ECL chemiluminescent detection system (Merck Millipore). ImageJ (National Institutes of Health) was used for the quantitative analysis. The experiments were repeated more than three times.

### Drug treatments and adoptive T cell transfer in E.G7-bearing mouse model

2×10^6^ E.G7 cells were inoculated subcutaneously into each C57BL/6 male mouse. When the average tumor size reached about 100 mm^3^, mice were randomly divided into seven groups (n = 6 per group): vehicle (control), 4 mg/kg Dox (Dox), 10 mg/kg Tax (Tax), 2×10^6^ CD8+ T-cells (2T), 5×10^6^ CD8+ T-cells (5T), 4 mg/kg Dox plus 2×10^6^ CD8+ T-cells (Dox+2T) and 10 mg/kg Tax plus 2×10^6^ CD8+ T-cells (Tax+2T). Single dose of 4 mg/kg Dox or 10 mg/kg Tax was administered *via i.p.* injection, and transferred OT-1 CD8+ T-cells were given by *i.v.* injection. For the combination therapy, Dox or Tax was given 24 hours before CD8+ T-cells transfer (see Figure 4A for details). Tumor volumes were measured with a caliper and calculated by the formula: length x width^2^ x 0.523. The experiments were repeated three times.

### Electrophoretic mobility shift assay (EMSA)

Nuclear proteins were isolated from cells or tumor tissues using Nuclear Extraction Kit (Chemicon International). The NF-κB/DNA binding activity was evaluated using the LightShift Chemiluminescent EMSA kit (Pierce). The isolation and analysis were performed using protocols provided by the manufacturer. The following DNA sequences were synthesized for EMSA analysis: AGTTGAGGGGACTTTCCCAGGC (sense) and GCCTGGGAAAGTCCCCTCAACT (antisense). Briefly, nuclear extracts were incubated with the biotin-labeled DNA probe for 20 min at room temperature. The DNA-protein complex was separated from free oligonucleotides on a 5% polyacrylamide gel, then transferred to a nylon membrane and cross-linked by UV light. The membranes were further incubated with streptavidin-horseradish peroxidase, and detected by enhanced ECL. The imageJ software was used for the quantitative analysis. The experiments were repeated three times.

### Enzyme-linked immunosorbent assay (ELISA)

Circulating bloods were drawn from the facial vein on day 3 after one low-dose Dox or Tax treatment, and serum samples were prepared by centrifugation at 2000 rpm for 10 min post coagulation. The level of serum IL-12 was determined by IL-12 ELISA kit (R&D Systems).

### Bioluminescent imaging (BLI) *in vivo*

Mice (*n* = 6 per group) receiving *pGBeLT*-transduced CD8+T cells were *i.p.* injected with 150 mg/kg D-luciferin for 15 min, then anesthetized with 1-3% isoflurane for imaging. The emitted photons were detected by the IVIS50 Imaging System (Xenogen) for 5 min. The Living Image software (Version 2.20, Xenogen) was used to quantify the signals emitted from the regions of interest (ROIs).

### Immunohistochemistry

Tumors removed from mice were frozen in Tissue-Tek OCT compound (Sakura Finetek) and sectioned with 10 μm thickness, and mounted on glass slides. Sections were incubated with 3% H_2_O_2_ for 10 min to quench the endogenous peroxidase activity, then rinsed with PBS. Slides were incubated with primary antibodies against CD8 and FOXP3 at 4°C overnight, developed with diaminobenzidine tetrahydrochloride (DAB), and finally counterstained with hematoxylin. All the staining procedures were followed the protocols provided by the manufacturer of IHC Select HRP/DAB kit (Millipore). CD8+ cells were defined as CTLs, and FOXP3+ cells were defined as Tregs. Slides were imaged with Olympus BX 61 microscope equipped with color CCD under 100 x magnification. Five slides were imaged and analyzed per group.

### Statistical analysis

All data were shown as the mean ± standard error. Student's *t*-test was used for the comparison between two groups. One-way ANOVA followed by Tukey's post hoc test was used when comparing more than two groups. Difference between the means were considered significant if *p* < 0.05 or less.

## SUPPLEMENTARY MATERIAL FIGURES


