# Supplementary material for: Enhancement of adoptive T cell transfer with single low dose pretreatment of doxorubicin or paclitaxel in mice
Source: Oncotarget. 2015 Dec 16;6(42):44134–50. doi: 10.18632/oncotarget.6628 (PMC4792547; doi:10.18632/oncotarget.6628)
Supplement: Supplementary file 1 [file oncotarget-06-44134-s001.pdf]

Enhancement of adoptive T cell transfer with single low dose pretreatment of doxorubicin or paclitaxel in mice

Supplementary Material

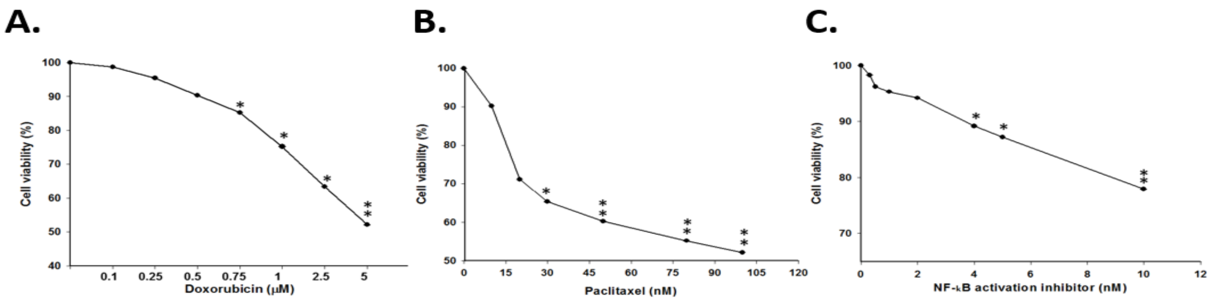

Supplementary Figure 1. The cytotoxic effects of Dox, Tax and QNZ in E.G7 cells.

(**A-C**) $1x10^5$ E.G7 cells/well plated in 96-well plate were treated with various doses of Dox, Tax and QNZ for 24 hours, and the cell viability was assayed by AlamarBlue assay. Cell viabilities at 90% for Dox and Tax treatments were 0.4 μM and 12.5 nM, respectively.

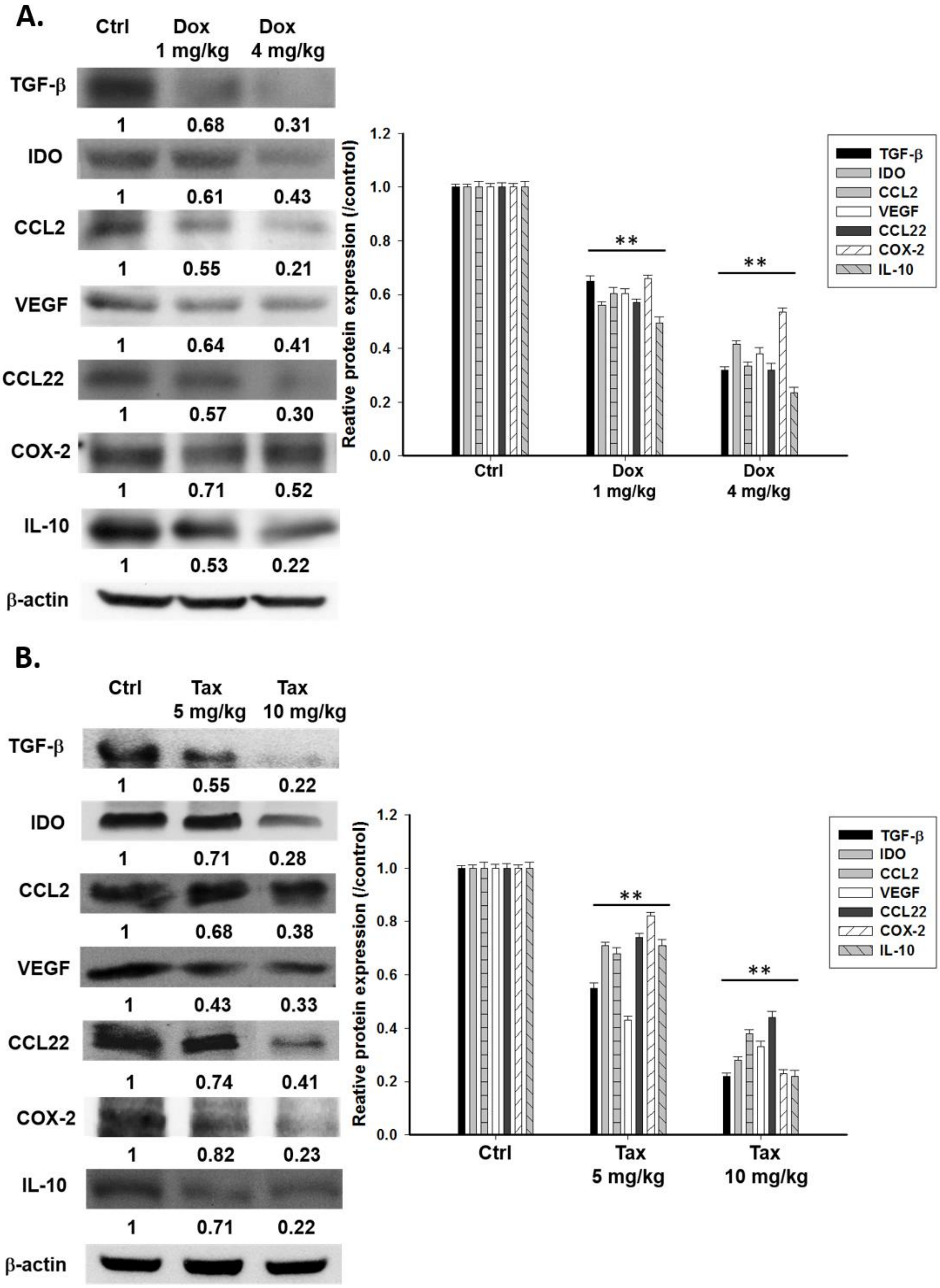
A.
Ctrl
Dox 1 mg/kg
Dox 4 mg/kg
TGF-β
1 0.68 0.31
IDO
1 0.61 0.43
CCL2
1 0.55 0.21
VEGF
1 0.64 0.41
CCL22
1 0.57 0.30
COX-2
1 0.71 0.52
IL-10
1 0.53 0.22
β-actin
Reative protein expression (/control)
1.2
1.0
0.8
0.6
0.4
0.2
0.0
**
**
Ctrl
Dox 1 mg/kg
Dox 4 mg/kg
TGF-β
IDO
CCL2
VEGF
CCL22
COX-2
IL-10
B.
Ctrl
Tax 5 mg/kg
Tax 10 mg/kg
TGF-β
1 0.55 0.22
IDO
1 0.71 0.28
CCL2
1 0.68 0.38
VEGF
1 0.43 0.33
CCL22
1 0.74 0.41
COX-2
1 0.82 0.23
IL-10
1 0.71 0.22
β-actin
Reative protein expression (/control)
1.2
1.0
0.8
0.6
0.4
0.2
0.0
**
**
Ctrl
Tax 5 mg/kg
Tax 10 mg/kg
TGF-β
IDO
CCL2
VEGF
CCL22
COX-2
IL-10

C.

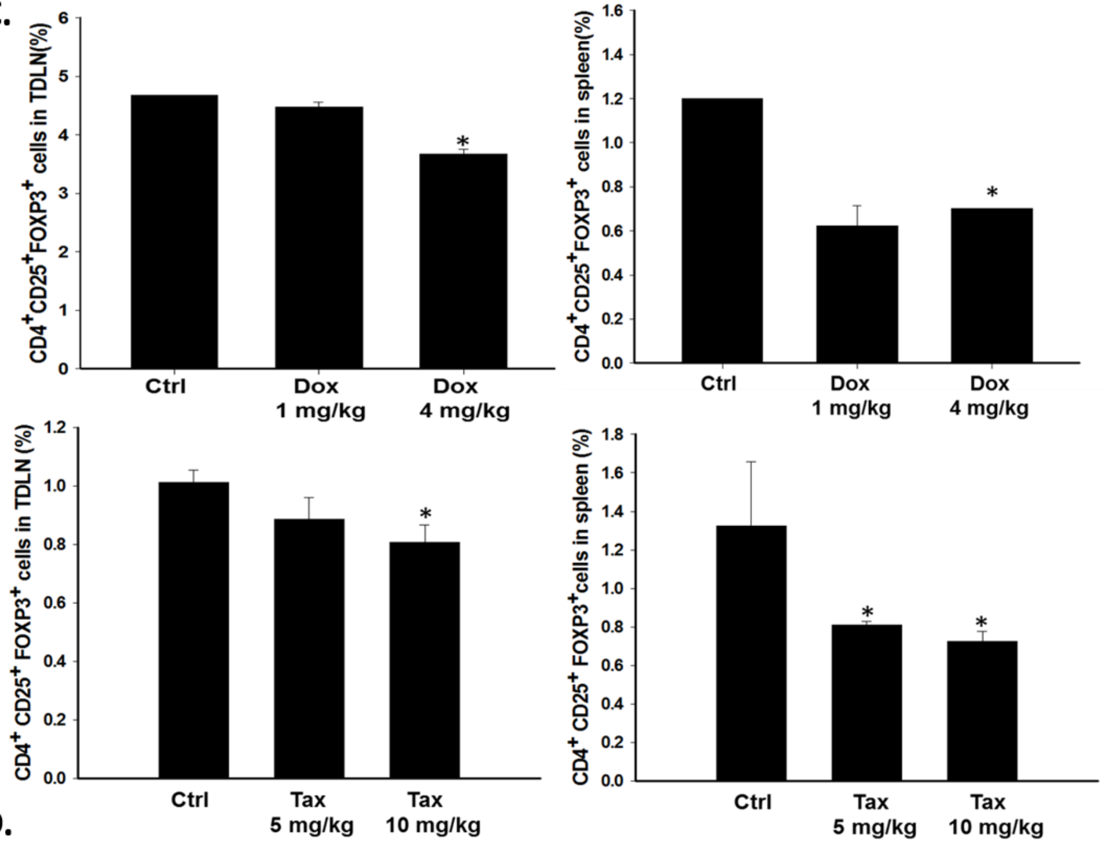

CD4+CD25+FOXP3+ cells in TDLN(%)
CD4+CD25+FOXP3+ cells in spleen(%)
CD4+ CD25+ FOXP3+ cells in TDLN (%)
CD4+ CD25+ FOXP3+cells in spleen (%)
Ctrl
Dox 1 mg/kg
Dox 4 mg/kg
Tax 5 mg/kg
Tax 10 mg/kg

D.

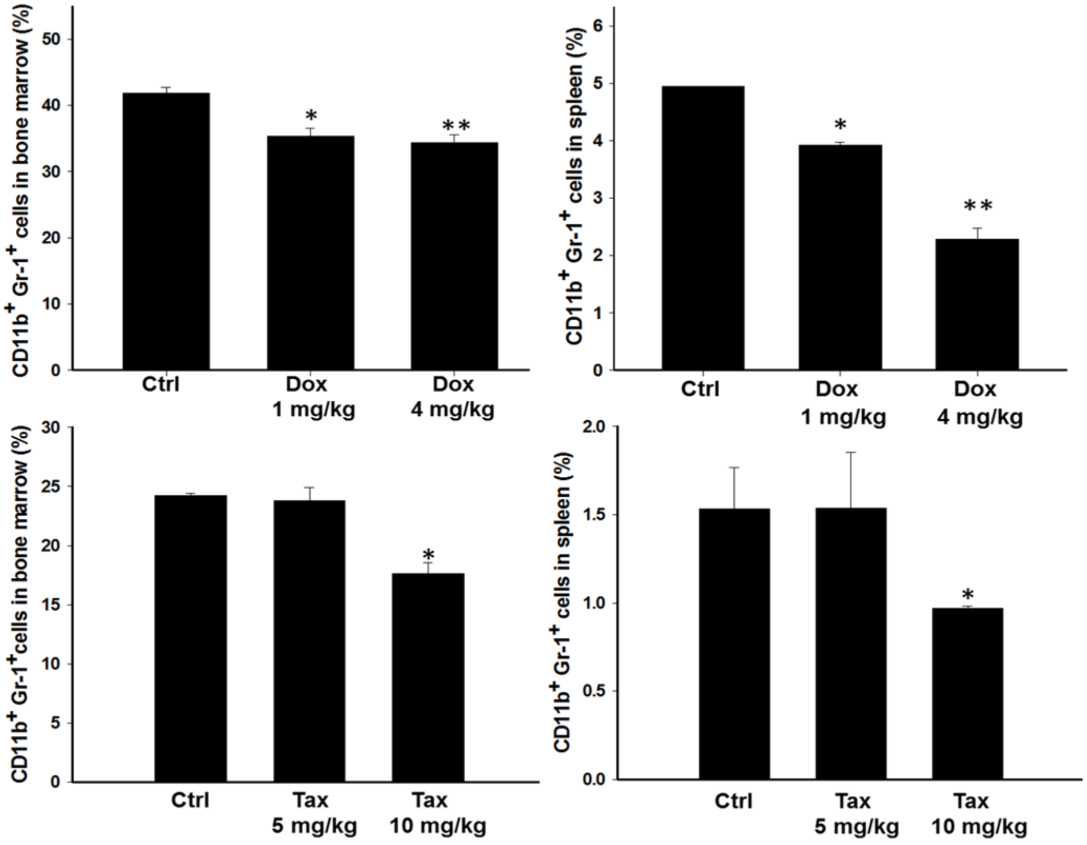

CD11b+ Gr-1+ cells in bone marrow (%)
CD11b+ Gr-1+ cells in spleen (%)
CD11b+ Gr-1+cells in bone marrow (%)
Ctrl
Dox 1 mg/kg
Dox 4 mg/kg
Tax 5 mg/kg
Tax 10 mg/kg

Supplementary Figure 2. Tumor microenvironment was slightly modified one day after single low-dose Dox or Tax treatment. (**A-B**) The expressions of immunosuppressive factors such as TGF-β, IDO, CCL2, VEGF, CCL22, COX-2 and IL-10 in tumors were significantly reduced in Dox- and Tax-treated groups analyzed by Western blot. (**C**) Percentages of Tregs in the spleen and tumor drained lymph nodes (TDLNs) were slightly decreased in groups treated with single low-dose Dox or Tax. (**D**) Percentages of MDSCs in the spleen and bone marrow were significantly decreased in groups treated with single low-dose Dox or Tax. * $p < 0.05$, ** $p < 0.01$, * $p < 0.05$ compared with that of the control.
